# Net Benefit of Anticoagulation in Subclinical Device-Detected Atrial Fibrillation

**DOI:** 10.1001/jamanetworkopen.2025.8461

**Published:** 2025-05-02

**Authors:** Aleksi K. Winstén, Ville Langén, K. E. Juhani Airaksinen, Konsta Teppo

**Affiliations:** 1 Department of Mathematics and Statistics, University of Turku, Turku, Finland; 2 Division of Medicine, Turku University Hospital and University of Turku, Turku, Finland; 3 Turku University Hospital and University of Turku, Turku, Finland; 4 Heart Centre, Turku University Hospital, Turku, Finland; 5 Faculty of Medicine, University of Turku, Turku, Finland; 6 Department of Life Technologies, University of Turku, Turku, Finland

## Abstract

**Importance:**

The role of anticoagulation for stroke prevention in patients with device-detected atrial high-rate episodes, also known as subclinical atrial fibrillation (AF), is a subject of equipoise.

**Objective:**

To assess the net benefit of nonvitamin K antagonist oral anticoagulants (NOACs) in patients with device-detected subclinical AF.

**Design, Setting, and Participants:**

Decision analytical model run with 10 000 patients with anticoagulation and 10 000 patients without anticoagulation in a clinical scenario of deciding whether to start NOACs for stroke prevention in patients with subclinical AF. A Markov decision model was conducted on October 1, 2024, to estimate net outcomes of NOACs. The patients had stroke risk and bleeding risks similar to those of patients in randomized trials of anticoagulation in subclinical AF.

**Exposure:**

Anticoagulation was modeled to decrease the risk of ischemic stroke by 32% and increase the risk of major bleeding by 62%. In probabilistic sensitivity analyses, the 95% CIs for treatment effect sizes were also considered.

**Main Outcomes and Measures:**

The main outcome measure for overall net benefit was the cumulative quality-adjusted life-years (QALYs) during the simulation. The model considered the number and severity of ischemic strokes, hemorrhagic strokes, other intracranial bleeds, and extracranial bleeds, as well as the number of deaths during a 10-year simulation.

**Results:**

When comparing the 2 cohorts of 10 000 patients (mean age, 77 years; 3700 [37%] women), those receiving NOAC therapy had 233 fewer ischemic strokes (21.7%), 55 fewer deaths (1.1%), and 453 more major bleeding events (37.3%) over a 10-year simulation period. Per patient, these differences translated to approximately 1 additional quality-adjusted week of life (0.024 QALYs) with NOAC treatment during the 10-year simulation. When the 95% CIs of treatment effect sizes were considered in probabilistic sensitivity analysis, there was a 65.8% probability that NOAC treatment leads to more QALYs than withholding treatment.

**Conclusions and Relevance:**

In this analytical model study, initiating NOACs in patients with device-detected subclinical AF was associated with a minimal increase in QALYs. However, the benefits were uncertain, and the effect size of the overall net benefit does not appear to be clinically meaningful.

## Key Points

**Question:**

Does anticoagulation provide a net benefit in patients with device-detected subclinical atrial fibrillation?

**Findings:**

In this analytical model study, nonvitamin K antagonist oral anticoagulant (NOAC) therapy in patients with subclinical atrial fibrillation resulted in a net benefit of approximately 1 additional week of quality-adjusted life per patient. When uncertainty in treatment effects was considered, there was only a 66% probability that NOAC treatment would result in more quality-adjusted life than withholding treatment.

**Meaning:**

These findings suggest that net benefit of anticoagulation for device-detected subclinical atrial fibrillation is uncertain, and the effect size is not clinically meaningful.

## Teaser

This decision analytical model study evaluates the net benefit of nonvitamin K antagonist oral anticoagulants in patients with device-detected subclinical atrial fibrillation.

## Introduction

Oral anticoagulation, either with vitamin K antagonists or nonvitamin K antagonist oral anticoagulants (NOACs), has been shown to effectively reduce the risk of ischemic stroke in patients with atrial fibrillation (AF).^1,2^ This evidence in stroke prevention is derived from studies conducted among patients with clinical AF, in whom symptoms have usually prompted the diagnosis of arrhythmia using 12-lead electrocardiography. However, contemporary cardiac implantable electronic devices and electronic wearables are able to monitor heart rhythm continuously and can therefore detect shorter asymptomatic episodes of AF that would not have previously come to clinical attention. This arrhythmia entity of asymptomatic atrial high-rate episodes is often referred to as subclinical AF. Optimal approach for its management, particularly regarding the role of oral anticoagulation, has remained unclear.

Recently, 2 randomized trials, Non–Vitamin K Antagonist Oral Anticoagulants in Patients With Atrial High Rate Episodes (NOAH-AFNET 6) and Apixaban for the Reduction of Thrombo-Embolism in Patients With Device-Detected Sub-Clinical Atrial Fibrillation (ARTESiA), evaluated the benefit of NOACs in patients with device-detected subclinical AF. The NOAH-AFNET 6 was halted prematurely due to safety concerns with a 2-fold increase in major bleeding with NOAC therapy and no statistically significant difference in stroke rates.^3^ In contrast, the ARTESiA trial reported that anticoagulation significantly reduced the risk of ischemic stroke at the expense of a higher bleeding rate.^4^ The absolute rate of stroke was lower than expected in both trials, at approximately 1 stroke per 100 patient-years. While initially the results of these 2 studies appeared to be discordant, their study-level meta-analysis demonstrated that, in fact, the treatment effect was consistent between the trials and reported a 32% decrease in the risk of ischemic stroke with NOACs, with a tradeoff of a 62% higher risk of major bleeding.^5^ Prior observational studies have reported increasing stroke rates with longer subclinical arrhythmia episodes and higher stroke risk scores, but substudies of the 2 randomized trials have not found robust thresholds for the burden of subclinical AF, nor for stroke risk scores, above which the efficacy and safety profile of anticoagulation would be significantly more favorable.^6,7,8,9^

Although the NOAH-AFNET 6 and ARTESiA trials have elucidated the prognosis of subclinical AF and the effects of its treatment, many clinically relevant questions remain unanswered. The stroke risk in patients with subclinical AF appears to be low, and determining if the one-third reduction of this already low stroke risk is worth the two-thirds increase in major bleeding can be challenging for clinicians. Numerical comparison of event rates does not capture the true burden of outcomes, as the severity of events varies considerably. Furthermore, anticoagulation modifies event severity, reducing the severity of ischemic strokes while increasing the severity of intracranial bleeding events.^4,10,11^ In addition to assessing whether there in fact even is a net benefit or net harm with anticoagulation, the magnitude of this effect remains unclear. Intuitively weighing the impact of possible outcomes on an individual’s life is complex, let alone effectively communicating this information to enable shared decision-making on the initiation of a new, somewhat costly, and potentially cumbersome medication. We aimed to answer these important clinical questions with a Markov decision model analysis. Based on the new trial-based data on outcome probabilities and prior evidence on their impact on quality of life, we assessed the outcomes of initiating NOACs in patients with device-detected subclinical AF.

## Methods

### Analytic Approach

The study follows, in applicable parts and excluding the economic aspects, the Consolidated Health Economic Evaluation Reporting Standards (CHEERS) reporting guideline statement. According to Finnish legislation on medical research, ethical approval and informed consent were deemed unnecessary, as this study used an analytical model based on publicly available data without collecting new data or accessing patient information. We estimated the net changes of the decision to initiate NOACs on the quality-adjusted life-years (QALYs) of a patient with subclinical AF, compared with the decision to withhold anticoagulation using a decision analytic Markov model. This modeling approach is necessary since the original trials did not report quality-of-life outcomes, and assessing net benefit using this measure in a clinical trial would require a substantially larger sample size and longer follow-up to reliably capture the long-term quality-of-life effects of the treatment. The Markov model consists of multiple health states individuals can move between based on specific transition probabilities (Table 1).

**Table 1.** Markov Model Input Parameters

**Input parameter**	**Estimate**	**Source**
Event rate, untreated rate per 100 patient-years		
Ischemic stroke	1.05^a^	Kirchhof et al,^3^ 2023; Healey et al,^4^ 2023; and McIntyre et al,^5^ 2023
Major bleeding	1.06^a^	
Hemorrhagic stroke	0.18^b^	
Other intracranial bleeding	0.15^b^	
Extracranial major bleeding	0.73^c^	
Death	4.26^a^	
Clinical atrial fibrillation	7.50^a^	
Outcome of anticoagulation, RR (95% CI)		
Ischemic stroke	0.68 (0.50-0.92)	McIntyre et al,^5^ 2023
Major bleeding	1.62 (1.05-2.50)	
Event severity, probability without NOAC (with NOAC)		
Ischemic stroke		
Death	0.187 (0.120)	Sennfält et al,^12^ 2019; Vinding et al,^13^ 2022; Healey et al,^4^ 2023; Walraven et al,^14^ 2002; Fang et al,^15^ 2012; and Diener et al,^16^ 2024
Severe disability	0.156 (0.124)	
Moderate disability	0.220 (0.247)	
Mild disability	0.437 (0.509)	
Hemorrhagic stroke		
Death	0.175 (0.305)	Rosand et al,^11^ 2004; Fang et al,^17^ 2007; Healey et al,^4^ 2023; Giugliano et al,^18^ 2013; Skaistis et al,^19^ 2015; and Toyoda et al,^20^ 2022
Severe disability	0.172 (0.206)	
Moderate disability	0.301 (0.190)	
Mild disability	0.352 (0.293)	
Other intracranial bleeding		
Death	0.157 (0.176)	Fang et al,^15^ 2012; Weimer et al,^21^ 2017; Poon et al,^22^ 2021; Giugliano et al,^18^ 2013; Healey et al,^4^ 2023; Skaistis et al,^19^ 2015; and Gaist et al,^23^ 2017
Severe disability	0.179 (0.237)	
Mild disability	0.332 (0.293)	
No disability	0.332 (0.293)	
Extracranial major bleeding		
Death	0.025 (0.035)	Fang et al,^17^ 2007; Walraven et al,^14^ 2002; Vora et al,^24^ 2020; Chornenki et al,^25^ 2023; Giugliano et al,^18^ 2013; Healey et al,^4^ 2023; Skaistis et al,^19^ 2015; and Gómez-Outes et al,^26^ 2021
Severe disability	0.008 (0.007)	
Mild disability	0.058 (0.056)	
No disability	0.909 (0.902)	
Base case quality of life, weight		
Well without events from age 77 to 80	0.794	Burström et al,^27^ 2006
Well without events from age 80 to 87	0.733	
Quality-adjusted life year, ratio		
Mild disability until 6 mo after event	0.88	Luengo-Fernandez et al,^28^ 2013
Mild disability from 6 mo after event	0.89	
Moderate disability until 6 mo after event	0.60	
Moderate disability from 6 mo after event	0.73	
Severe disability until 6 mo after event	0.16	
Severe disability from 6 mo after event	0.45	
Death	0	
Mortality within the first year after events, 1-y probability		
After ischemic stroke	0.14	Sennfält et al,^12^2019
After any intracranial bleeding	0.16	
Event risks after developing clinical AF, 1-y probability		
Ischemic stroke	0.025	Teppo et al,^29^ 2022
Hemorrhagic stroke	0.005	
Other intracranial bleeding	0.005	
Extracranial major bleeding	0.028	
Death	0.110	

Abbreviations: AF, atrial fibrillation; NOAC, nonvitamin K antagonist oral anticoagulant; RR, relative risk.

^a^ Average rate from the NOAH-AFNET 6 and ARTESiA trials.

^b^ Derived from the ARTESiA trial.

^c^ Calculated by subtracting the intracranial bleeding rate from the total major bleeding rate.

Rates and probabilities were transformed to 1-month probabilities to the model.

### Base Case Patient

As a base case patient for the analysis, we used a patient aged 77 years, aligning with the mean age of the trial populations, and applied the average untreated stroke and bleeding rates from the NOAH-AFNET 6 and ARTESiA trials.^3,4^ Similarly, average mortality rates were obtained from these trials. Our model focused on ischemic strokes, major bleeding, and mortality since these are the main outcomes to consider when evaluating the benefits and harms of anticoagulant therapy. Other events, such as myocardial infarctions and pulmonary embolisms, were excluded from the model also because their incidence did not change with anticoagulation in the trials, and their impact on quality of life varies considerably.

### Outcomes Associated With Anticoagulation

The pooled point risk estimates of the meta-analysis combining the 2 trials were used as the effect sizes for anticoagulation on stroke and major bleeding.^5^ The NOAH-AFNET 6 trial did not report rates for specific bleeding subtypes, and the ARTESiA trial did not observe a statistically significant increase in intracranial bleeding with anticoagulation. However, both trials were underpowered to detect significant differences in the bleeding subtypes, and the interpretation of the bleeding risk estimates of the ARTESiA trial is hampered since it had aspirin as the comparator group instead of placebo.^30^ That said, in addition to the point estimates of the ARTESiA trial, there are signals that nonintracranial bleedings predominate in the bleeding risks associated with NOACs, although placebo-controlled data in this regard are lacking.^1^ Therefore, in the main analyses, we assigned an 80% weight to nonintracranial bleedings for the increase in bleedings caused by NOACs, maintaining the overall sum of increased major bleedings consistent with the results of the meta-analysis (ie, an overall 62% increase in major bleedings with anticoagulation, of which 80% were nonintracranial bleedings). The outcome of different weights was tested in the sensitivity analyses. Following all major bleeding events in patients receiving anticoagulation therapy, anticoagulation was temporarily paused for 1 month in the model.

Anticoagulation was not observed to significantly affect mortality in the 2 trials nor in their meta-analysis, and thus the same mortality rates were used for the anticoagulation and nonanticoagulation groups of the model.^5^ The initial 30-day mortality related to stroke and bleeding events was included in the model, and additionally, ischemic stroke and all intracranial bleeding events were considered to increase mortality for the first year following the event, with these mortality probabilities derived from a large Swedish population-based study.^12^ Probabilities for the severity of stroke and bleeding events in the anticoagulation and nonanticoagulation groups of the model were approximated from previous observations in patients with and without anticoagulation, as well as from the data provided by the NOAH-AFNET 6 and ARTESiA trials (Table 1). To address the variability in previously reported event severity probabilities, the least squares method was used to estimate severity probabilities that best fit the values from prior studies.

### Development of Clinical AF

Patients with subclinical AF often develop clinical AF, and their subsequent treatment is guided by the existing clinical practice guidelines on the management of AF, usually consisting of anticoagulation. In our model, all these patients with overt AF were considered to have the average stroke, bleeding, and mortality rates of real-life contemporary patients diagnosed with clinical AF, irrespective of whether they were anticoagulated in the model before developing clinical AF.^29^ Thus, after the onset of clinical AF, the prognosis was computed similarly in the model for both decision groups. In patients with clinical AF, we applied event severity probabilities of the patients who are treated with anticoagulation.

### Utility Weights

The net benefit outcome in our study was assessed in terms of QALYs, where clinical events reduced patients’ quality of life based on the type and severity of the event according to previously published quality of life data. The baseline QALY weights were derived from age-specific utility values of the general Swedish population provided by Burstrom et al.^27^ To address the quality-of-life outcomes from ischemic strokes and intracranial hemorrhages, the individual’s QALY values changed based on the study by Luengo-Fernandez et al,^28^ which details the quality of life of patients with stroke according to the event’s severity. In our model, new QALY weights were calculated by multiplying an individual’s pre-event QALY weight by the ratio of the QALY in patients with the event severity of interest to that of control patients as reported in the study. For the rare major extracranial bleeding events that resulted in permanent disability, we applied the same QALY ratios according to the severity of the disability. The QALY weights and ratios used in the model are presented in Table 1.

### Data Analysis

The model used a 1-month cycle length, and all previously mentioned figures were transformed into 1-month probabilities (Table 1). The simulation was run for a 10-year period with 10 000 samples in both decision groups. As the primary outcome measure, cumulative QALYs were compared between individuals initially chosen to start anticoagulant therapy and those who were not. The cumulative number of outcome events and life-years were also counted. eFigure 1 in Supplement 1 depicts the Markov model and the health states. The used event rates and probabilities, along with their literature sources, are presented in Table 1. The modeling was conducted on October 1, 2024. In the interest of research reproducibility, we have deposited the codes of the Markov model in the Zenodo repository.^31^ All analyses were performed with R version 4.2.2 (R Project for Statistical Computing).

Sensitivity analyses were performed to address parameter uncertainty of the model. In the main analysis, the effect of NOACs on stroke and bleeding risk was computed according to the point estimates of the meta-analysis of the NOAH-AFNET 6 and ARTESiA trials (relative risks of 0.68; 95% CI, 0.50-0.92 and 1.62; 95% CI, 1.05-2.50, respectively).^5^ In probabilistic sensitivity analyses, the 95% CIs of these estimates were also considered, assuming that they follow a log-normal distribution. The model was run for a 10-year period with 2000 iterations of sampled risk estimates from this log-normal distribution, with 10 000 patients in both decision groups. The mean QALY difference and the proportion of the iterations leading to incremental QALYs in favor of NOACs was calculated.

Moreover, we investigated how varying model inputs across a clinically relevant scale was associated with the difference in cumulative QALYs between the decision groups over a 10-year simulation period. First, while randomized clinical trials are the criterion standard in determining relative treatment effect, the absolute effect size may be different in patients treated outside the clinical setting due to usually higher untreated risks when compared with trial participants. Thus, to assess potential changes in the effect size, the initial ischemic stroke, bleeding, and mortality rates were raised to reflect observational studies (1.9, 1.7, and 8.5 per 100 patient-years, respectively).^32,33^ Second, we explored different weights for the outcome of NOACs on bleeding types, ranging from 70% to 90% weight for the proportion of nonintracranial bleedings of the overall increase in major bleedings. Third, we adjusted the effects of NOACs on stroke and bleeding based on the hazard ratio point estimates of the trials: 0.79 for ischemic stroke and 2.10 for major bleeding in NOAH-AFNET 6 and 0.62 for ischemic stroke and 1.36 for major bleeding in ARTESiA.

Additionally, a substudy of the ARTESiA trial showed a trend toward better stroke prevention with NOACs in patients with higher stroke risk scores.^9^ However, this trend was not statistically significant, and a similar phenomenon was not observed in the NOAH trial.^7^ Nonetheless, as a hypothesis-generating analysis, we performed separate analyses to explore the net benefit in the 3 CHA_2_DS_2_-VASc (congestive heart failure [1 point], hypertension [1 point], age ≥75 years [2 points], diabetes [1 point], history of stroke or transient ischemic attack [2 points], vascular disease [1 point], age 65-74 years [1 point], female sex category [1 point]) score categories of the ARTESiA substudy (>4, 4, or >4). We used the average nonanticoagulated stroke and major bleeding rates of the ARTESiA and NOAH trials and the hazard ratio point estimates for stroke and bleeding from the ARTESiA trial. Mortality rates in different risk score categories were not reported in the ARTESiA substudy, and they were derived from the NOAH trial.^7^ The values used in this sensitivity analysis are presented in the eTable in Supplement 1.

Finally, current guidelines recommend considering prior stroke and bleeding events, among other risk factors, when deciding on anticoagulant therapy for subclinical AF.^34,35^ Thus, as a sensitivity analysis, we constructed a separate model in which changes in treatment status after experienced events were accounted for: the occurrence of an intracranial bleeding event in a patient using NOACs led to cessation of anticoagulation, and an ischemic stroke event led to the initiation of anticoagulation. Cumulative QALYs in a 10-year model were compared based on the initial anticoagulation decision in an intention-to-treat manner.

## Results

**Figure 1.**
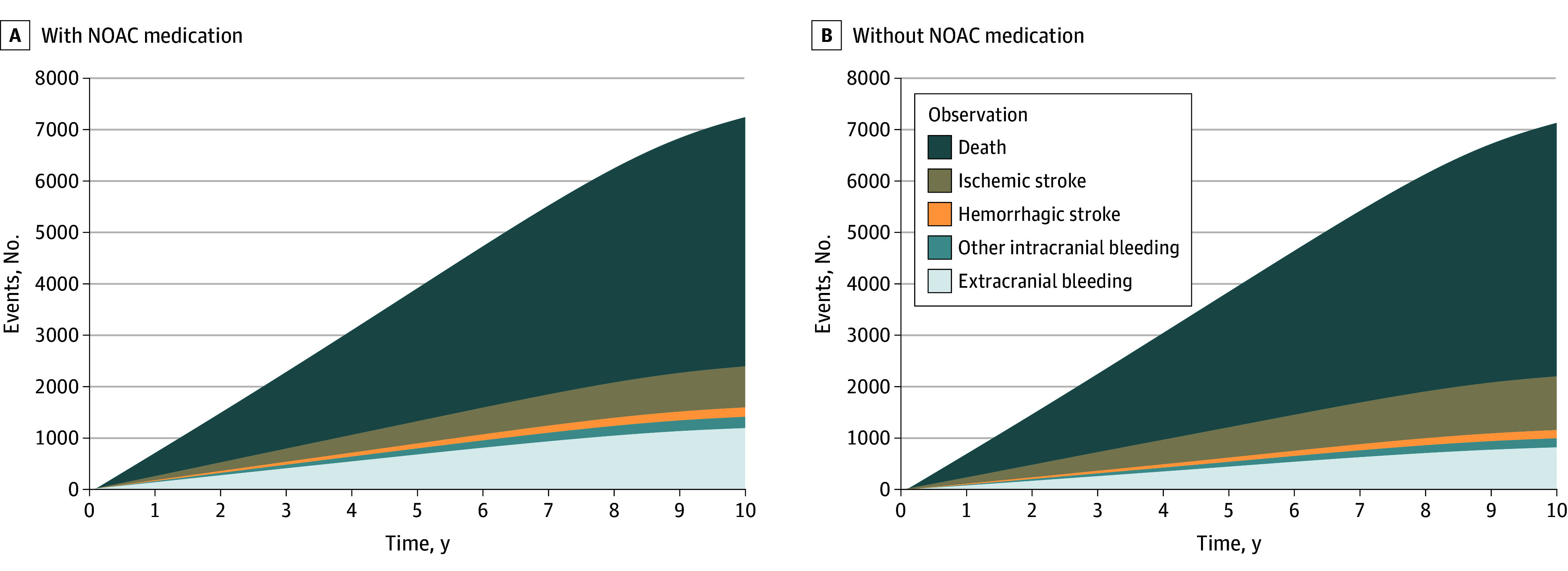
Incidence of Different Outcome Events During the 10-Year Simulation NOAC indicates nonvitamin K antagonist oral anticoagulant.

**Figure 2.**
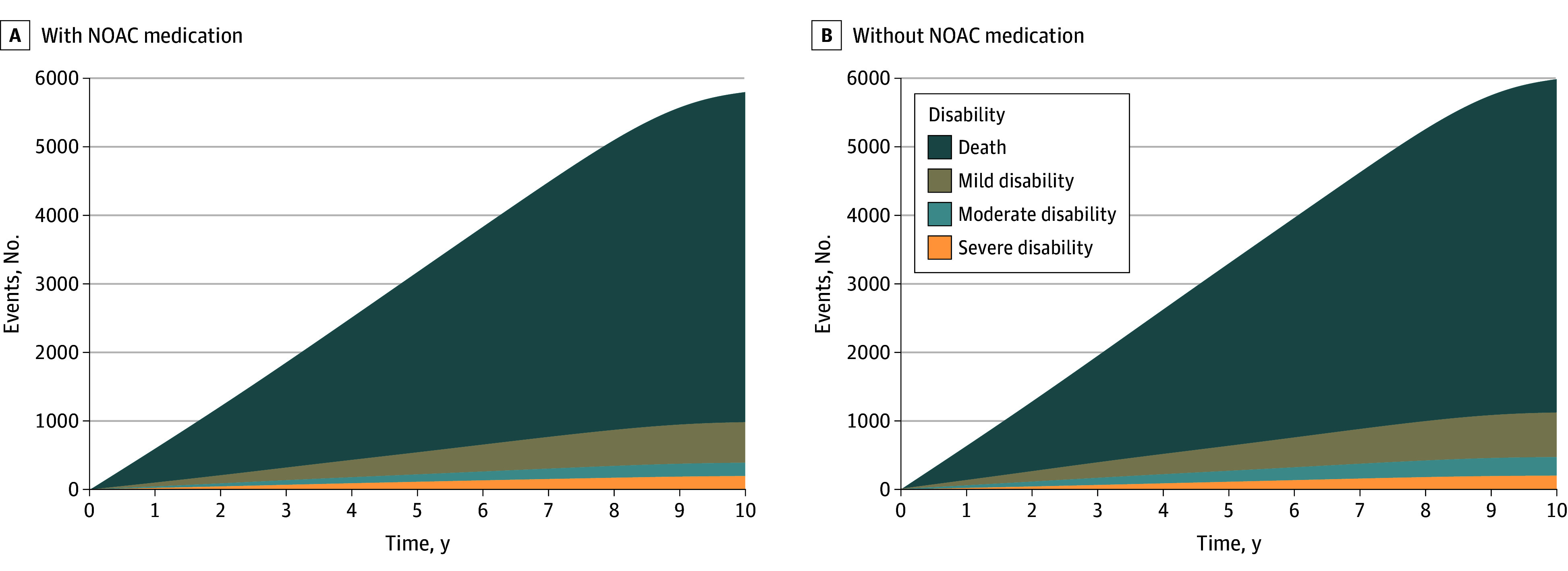
Incidence of Outcome Events According to Event Severity During the 10-Year Simulation Includes only events causing permanent disability, nondisabling events are not counted. NOAC indicates nonvitamin K antagonist oral anticoagulant.

**Figure 3.**
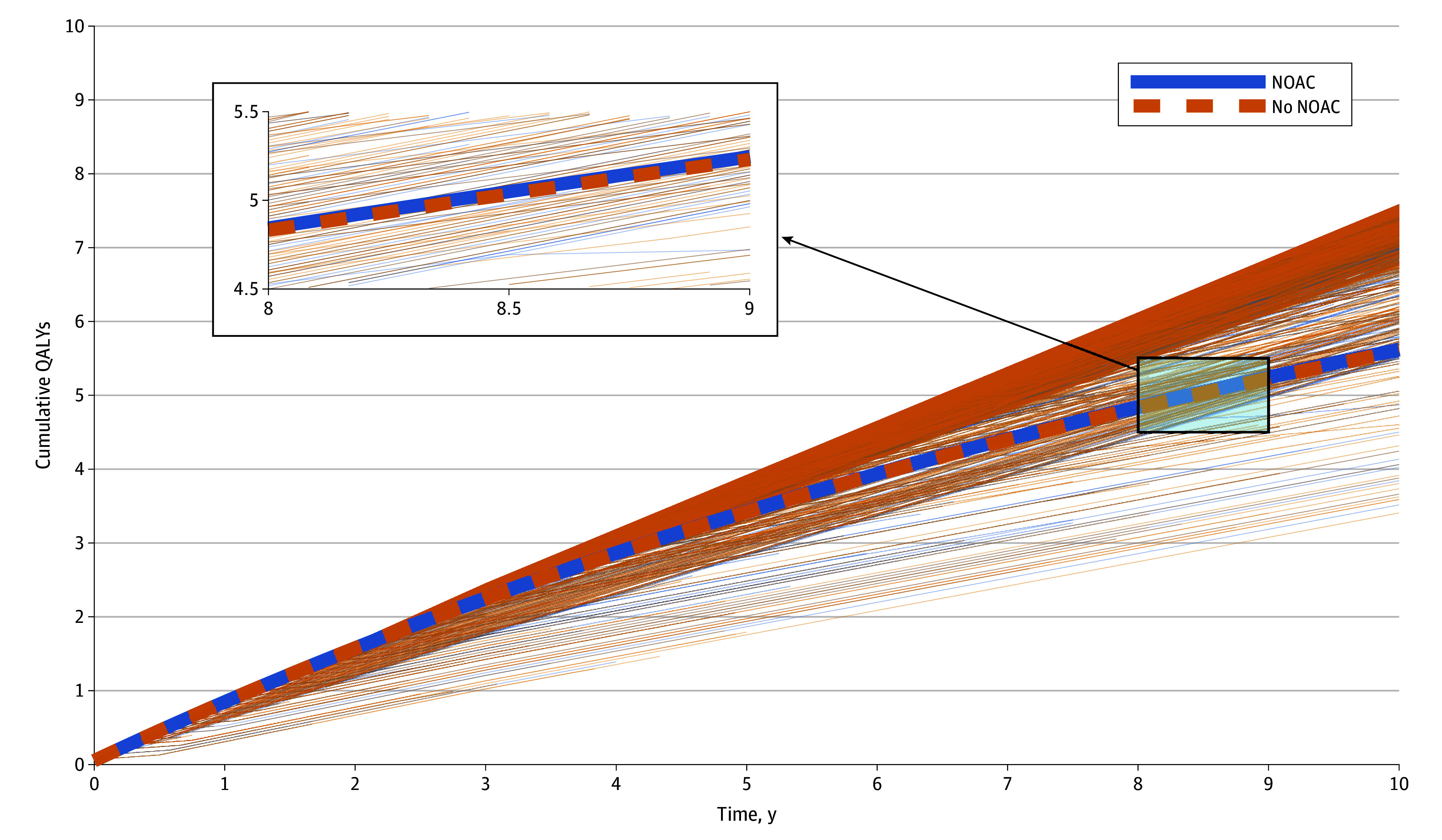
Cumulative Quality-Adjusted Life-Years Thick blue line represents the mean cumulative quality-adjusted life-years per patient with anticoagulation and the thick dashed yellow line the mean cumulative quality-adjusted life-years without anticoagulation. The thin lines in corresponding colors represent cumulative quality-adjusted life-years of individual patients with and without anticoagulation. NOAC indicates nonvitamin K antagonist oral anticoagulant.

In the base-case scenario, initiating NOACs for patients with device-detected subclinical AF resulted in 233 (21.7%) fewer ischemic strokes, 55 (1.1%) fewer deaths, and 453 (37.3%) more major bleeding events per 10 000 patients over a 10-year simulation period, when compared with withholding anticoagulation in the same-sized cohort (Figure 1, Table 2).^13,14,16,18,19,20,21,22,23,24,25,26^ In addition to the lower mortality, initiating NOACs resulted in slightly fewer events causing severe, moderate, or mild disability (Figure 2). Per patient, these differences translated to an additional 0.024 years of life and 0.024 QALYs with NOAC treatment during the 10-year simulation (Figure 3, Table 2). The mean time in subclinical AF (before death or progression to overt clinical AF) was 5.80 years in the NOAC treatment group and 5.78 years in the group without NOAC treatment.

**Table 2.** Model Outcomes for 10 000 Patients With and 10 000 Patients Without Nonvitamin K Antagonist Oral Anticoagulants (NOACs) During the 10-Year Simulation

Outcome	Therapy, No.		Difference^a^	Difference per patient^a^
	Without NOAC	With NOAC		
Ischemic stroke	1076	843	−233	−0.023
Major bleeding	1213	1664	453	0.045
Hemorrhagic stroke	170	192	22	0.002
Other intracranial bleeding	182	229	47	0.005
Extracranial bleeding	861	1243	382	0.038
Deaths	5179	5124	−55	−0.006
Life-years	74 928	75 168	240	0.024
QALYs	55 760	56 001	241	0.024

Abbreviation: QALY, quality-adjusted life year.

^a^ Differences were calculated as without NOAC minus with NOAC.

In the probabilistic sensitivity analysis, which accounted also for the 95% CIs of the effect sizes of NOACs on both stroke and bleeding, NOAC therapy resulted in higher QALYs during the 10-year period in 1316 (65.8%) of the 2000 iterations of risk estimates. The mean QALY difference in these simulations was 0.016 per patient in favor of NOAC therapy (eFigure 2 in Supplement 1).

When the initial untreated stroke, bleeding, and mortality rates were raised to levels reported in observational studies, NOAC therapy resulted in an incremental 0.030 QALYs per patient over the 10-year simulation. When the proportion of extracranial bleeding events among the increased bleeding events was varied from 70% to 90%, the QALY difference in favor of NOAC therapy ranged from 0.013 to 0.031 per patient, respectively. When the model was run with the risk estimates from the NOAH-AFNET 6 trial, NOAC therapy resulted in 0.013 fewer QALYs per patient over 10 years. With the risk estimates from ARTESiA, NOAC therapy led to 0.045 incremental QALYs per patient over 10 years. In the exploratory analyses stratified by the CHA_2_DS_2_-VASc score, NOAC therapy resulted in an increase of 0.023, 0.039, and 0.093 QALYs per patient in those with scores less than 4, equal to 4, and greater than 4, respectively. Finally, when dynamic changes in treatment status after ischemic stroke and intracranial bleeding events were incorporated in the model (ie, anticoagulation was stopped after intracranial bleeding events and started after an ischemic stroke) the initial decision to start anticoagulation at the beginning of the simulation led to 0.006 lower QALYs per patient when compared with withholding treatment.

## Discussion

The current study employed Markov decision modeling to evaluate the net benefit of treating patients with device-detected subclinical AF with NOACs. Over the 10-year simulation period, patients initiated with NOACs had fewer ischemic strokes and more major bleeding events, and these resulted in marginally higher total life-years and QALYs when compared with withholding treatment. However, importantly, the magnitude of outcome differences was small and unlikely to be clinically meaningful.

To our knowledge, this is the first study to model the net effect size of anticoagulation in patients with subclinical AF. Previous evidence on the overall benefits of NOACs in this patient group has not been entirely conclusive, with the results of the 2 major trials pointing in somewhat different directions. Additionally, disparities in the trial designs complicate the implementation of the evidence they provide.^5^ Current US guidelines emphasize the importance of personalized risk assessment and shared decision-making and recommend considering NOACs for patients with longer atrial high-rate episodes and high stroke risk scores.^34^ However, these recommendations are based on observational data and have not been strongly supported by the data from the 2 trials. Moreover, intuitively weighing the net impact of the treatment on patients’ lives in a clinical setting is challenging, particularly when the initial untreated risks are so low. To achieve this, it is crucial not only to compare event rates but also to assess the variations in mortality and quality of life associated with different events.^36^ When initiating a new medication with rare but serious adverse risks, it is essential to consider patients’ values and preferences, but effectively translating previous evidence to the patient to facilitate shared decision-making is difficult.

The most important measure in our results is the QALY difference, which at the 10-year point of the simulation corresponded approximately to 1 quality-adjusted week of life in favor of anticoagulation. Similarly, anticoagulation was associated with a marginal reduction in mortality, translating on average to only 9 days of life per patient over 10 years. Initiating anticoagulation in the subclinical phase of AF led to a 22% decrease in ischemic strokes and a 37% increase in major bleeding, including a 20% increase in intracranial bleeding. These estimates provide a clear view of the potential benefits and harms of anticoagulation, reflecting a relatively balanced net outcome. Moreover, the small potential gains, measured in life-years and QALYs, are considerably easier to communicate to patients than the metrics provided by previous studies.

In sensitivity analyses accounting for parameter uncertainty, the results were materially similar to those of the main analysis. Anticoagulation appeared beneficial when the model used the risk estimates from the ARTESiA trial, but slightly harmful when the risk estimates from the NOAH-AFNET 6 trial were applied. These results are concordant with the main results of these 2 trials and underscore the marginal effect size and uncertainty in the net benefit of anticoagulation. In the exploratory analyses across different CHA_2_DS_2_-VASc score categories, NOAC therapy was associated with a larger increase in QALYs for patients with a score greater than 4, corresponding to approximately 1 quality-adjusted month per patient. However, this finding should be interpreted with caution, as, in fact, none of the multiple interactions tested between treatment effects and risk categories in the ARTESiA and NOAH substudies reached statistical significance.^7,9^ When the initial stroke, bleeding, and mortality rates were raised to a level that better reflects data from observational studies, the QALY increase with NOACs was consistent with the main analysis, approximately 1 week per patient. Indeed, despite substantial changes in model parameters, the magnitude of the QALY difference remained small, ranging approximately from a decrease of 1 week to an increase of 1 month over 10 years. Importantly, when the 95% CIs of the treatment effects were considered in the probabilistic sensitivity analyses, there was only a 66% certainty that anticoagulation was associated with a positive net effect, with a mean effect size of 1 week. Overall, these results suggest that the question of whether or not to start NOACs for a patient with subclinical AF may actually have very limited clinical significance. It is also worth noting that the probabilistic sensitivity analysis indicated a 34% possibility that the treatment could be harmful to the patient.

The small effect size of the benefit of anticoagulation is understandable, given the low untreated stroke rate it aims to mitigate and the substantially higher competing risk of mortality in this patient population characterized by advanced age and high prevalence of comorbidities. The increase in bleeding events, although largely consisting of milder extracranial bleeds, further reduces the potential benefits of anticoagulation. Additionally, clinical AF can be considered a competing event. Once overt AF develops, the decision on anticoagulation becomes more straightforward according to current guidelines, making it outside the scope of the initial question of whether or not to start anticoagulation in the clinical setting of subclinical AF. Indeed, the rather short time before these competing events limits the likelihood that the small stroke risk will materialize for patients. The multistate Markov model used inherently incorporates these competing events and their outcomes. Moreover, the competing risks are even higher in real-life patients.

Questions remain as to what to recommend for patients and what patients would choose. Prior studies have shown that patients are generally more inclined to avoid strokes than bleeding events, even more so than their physicians, which is in part understandable since strokes result in permanent loss of brain tissue, whereas major bleeding is typically reversible.^37,38^ However, intracranial bleedings, despite their small share of all major bleeding events, actually confer substantial mortality and permanent disability, particularly with prior anticoagulantion.^15,17^ It may be that patients are less aware of potential harms of bleeding events, and thus the fear of strokes dominates patients’ views in treatment decisions. Moreover, patients’ values and preferences vary considerably; some are willing to pursue all possible treatments for even minor gains, while others prioritize avoiding potentially cumbersome medications, their costs, and even minor adverse effects.

However, we would argue that for practically all patients diagnosed with subclinical AF by a cardiac device, who are often well over the age of 80 years with several preexisting comorbidities, the potential gains from anticoagulation would seem negligible. Indeed, the uncertain benefits of additional days or weeks over a 10-year perspective would likely be considered minimal by most patients and not worth the burden of a new medication. Nevertheless, it is possible that some younger patients with longer life expectancies are more willing to initiate NOACs to benefit from the small potential gains in stroke reduction over a longer period. However, this is generally not the case for elderly patients with cardiac devices detecting subclinical AF in clinical practice.^39^ Of the 2 trials on NOACs in subclinical AF, our results support the conclusions of the NOAH-AFNET 6. Our results align with the cautious level IIb recommendation of the latest European guidelines on anticoagulation for device-detected AF and do not support upgrading this recommendation.^35^ The minimal effect size in our analysis is also concordant with the small and uncertain effect of AF screening on clinical outcomes.^40^ Indeed, in light of current evidence, it remains uncertain whether subclinical AF should even be routinely screened for with cardiac devices implanted for other indications.

### Limitations

The most important limitations of our study are related to the inherent challenges of mathematically modeling complex real-life scenarios. Parameter uncertainty may affect the results of the main analysis, since due to a lack of available data, we were unable to derive all model input values directly from randomized studies investigating patients with subclinical AF on NOACs. Relatedly, baseline risks for stroke, bleeding and mortality are most likely higher in real-life patients with subclinical AF than in the trial participants. However, importantly, all these aspects were explored in the sensitivity analyses, wherein the magnitude of differences between the treatment decisions remained small even when the model inputs were changed across a clinically credible range. Our model’s base-case scenario reflected average age and treatment effects from the NOAH-AFNET 6 and ARTESiA trials, but the benefits of anticoagulation may vary in some specific clinical scenarios, such as in younger patients with higher stroke risk scores. Of note, our analysis focused on stroke and major bleeding events, excluding nonmajor bleeding. Additionally, our study did not account for the impact of NOAC medication use per se on quality of life, such as associated costs, pharmacy visits, minor adverse effects, and the need for additional blood tests. The model also assumed complete adherence to initiated NOAC therapy. All these factors would likely further reduce the observed marginal benefits of anticoagulation in a real-world setting. Moreover, the utility weights used cannot account for all variations in subjective experiences and patient values. For instance, some patients may view a severely disabling stroke as a worse outcome than death.^41^ Finally, mathematical models cannot account for all the multifaceted possibilities that may occur for patients in real life; however, our model considered the most important aspects that the decision to initiate anticoagulation actually affects.

## Conclusions

In this decision analysis model study, initiating anticoagulation in patients with device-detected subclinical AF was associated with minimally higher QALYs. However, the benefits were uncertain, and the effect size did not appear to be clinically meaningful. Overall, the results do not support routine use of NOACs for stroke prevention in patients with device-detected subclinical AF.

## Notes

Supplement 1. **eTable. **Model input parameters used in the sensitivity analyses for patients with CHA2DS2-VASc scores of less than 4, 4, and more than 4 **eFigure 1.** Structure in the Markov decision model cycle. **eFigure 2.** Results of the probabilistic sensitivity analysis considering the uncertainty in the treatment effects for stroke and bleeding

Supplement 2.
Data Sharing Statement

## References

[1] Ruff CT, Giugliano RP, Braunwald E, . Comparison of the efficacy and safety of new oral anticoagulants with warfarin in patients with atrial fibrillation: a meta-analysis of randomised trials. Lancet. 2014;383(9921):955-962. doi:10.1016/S0140-6736(13)62343-0 24315724

[2] Hart RG, Pearce LA, Aguilar MI. Meta-analysis: antithrombotic therapy to prevent stroke in patients who have nonvalvular atrial fibrillation. Ann Intern Med. 2007;146(12):857-867. doi:10.7326/0003-4819-146-12-200706190-00007 17577005

[3] Kirchhof P, Toennis T, Goette A, ; NOAH-AFNET 6 Investigators; NOAH-AFNET6 sites and investigators. Anticoagulation with edoxaban in patients with atrial high-rate episodes. N Engl J Med. 2023;389(13):1167-1179. doi:10.1056/NEJMoa2303062 37622677

[4] Healey JS, Lopes RD, Granger CB, ; ARTESIA Investigators. Apixaban for stroke prevention in subclinical atrial fibrillation. N Engl J Med. 2024;390(2):107-117. doi:10.1056/NEJMoa2310234 37952132

[5] McIntyre WF, Benz AP, Becher N, . Direct oral anticoagulants for stroke prevention in patients with device-detected atrial fibrillation: a study-level meta-analysis of the NOAH-AFNET 6 and ARTESiA trials. Circulation. 2024;149(13):981-988. doi:10.1161/CIRCULATIONAHA.123.067512 37952187

[6] Becher N, Toennis T, Bertaglia E, . Anticoagulation with edoxaban in patients with long atrial high-rate episodes ≥24 h. Eur Heart J. 2024;45(10):837-849. doi:10.1093/eurheartj/ehad771 37956458 PMC10919916

[7] Lip GYH, Nikorowitsch J, Sehner S, . Oral anticoagulation in device-detected atrial fibrillation: effects of age, sex, cardiovascular comorbidities, and kidney function on outcomes in the NOAH-AFNET 6 trial. Eur Heart J. 2024;45(19):1733-1737. doi:10.1093/eurheartj/ehae225 38591192 PMC11107119

[8] McIntyre WF, Benz AP, Healey JS, . Risk of stroke or systemic embolism according to baseline frequency and duration of subclinical atrial fibrillation: insights from the ARTESiA trial. Circulation. 2024;150(22):1747-1755. doi:10.1161/CIRCULATIONAHA.124.069903 39229707

[9] Lopes RD, Granger CB, Wojdyla DM, . Apixaban vs aspirin according to CHA_2_DS_2_-VASc score in subclinical atrial fibrillation: insights from ARTESiA. J Am Coll Cardiol. 2024;84(4):354-364. doi:10.1016/j.jacc.2024.05.002 39019530

[10] Hylek EM, Go AS, Chang Y, . Effect of intensity of oral anticoagulation on stroke severity and mortality in atrial fibrillation. N Engl J Med. 2003;349(11):1019-1026. doi:10.1056/NEJMoa022913 12968085

[11] Rosand J, Eckman MH, Knudsen KA, Singer DE, Greenberg SM. The effect of warfarin and intensity of anticoagulation on outcome of intracerebral hemorrhage. Arch Intern Med. 2004;164(8):880-884. doi:10.1001/archinte.164.8.880 15111374

[12] Sennfält S, Norrving B, Petersson J, Ullberg T. Long-term survival and function after stroke: a longitudinal observational study from the Swedish Stroke Register. Stroke. 2019;50(1):53-61. doi:10.1161/STROKEAHA.118.022913 30580719

[13] Vinding NE, Kristensen SL, Rørth R, . Ischemic stroke severity and mortality in patients with and without atrial fibrillation. J Am Heart Assoc. 2022;11(4):e022638. doi:10.1161/JAHA.121.022638 35156393 PMC9245802

[14] van Walraven C, Hart RG, Singer DE, . Oral anticoagulants vs aspirin in nonvalvular atrial fibrillation: an individual patient meta-analysis. JAMA. 2002;288(19):2441-2448. doi:10.1001/jama.288.19.2441 12435257

[15] Fang MC, Go AS, Chang Y, . Thirty-day mortality after ischemic stroke and intracranial hemorrhage in patients with atrial fibrillation on and off anticoagulants. Stroke. 2012;43(7):1795-1799. doi:10.1161/STROKEAHA.111.630731 22539546 PMC3383879

[16] Diener HC, Becher N, Sehner S, ; NOAH-AFNET 6 investigators. Anticoagulation in patients with device-detected atrial fibrillation with and without a prior stroke or transient ischemic attack: the NOAH-AFNET 6 trial. J Am Heart Assoc. 2024;13(17):e036429. doi:10.1161/JAHA.124.036429 39190564 PMC11646511

[17] Fang MC, Go AS, Chang Y, . Death and disability from warfarin-associated intracranial and extracranial hemorrhages. Am J Med. 2007;120(8):700-705. doi:10.1016/j.amjmed.2006.07.034 17679129 PMC3534961

[18] Giugliano RP, Ruff CT, Braunwald E, ; ENGAGE AF-TIMI 48 Investigators. Edoxaban versus warfarin in patients with atrial fibrillation. N Engl J Med. 2013;369(22):2093-2104. doi:10.1056/NEJMoa1310907 24251359

[19] Skaistis J, Tagami T. Risk of fatal bleeding in episodes of major bleeding with new oral anticoagulants and Vitamin K antagonists: a systematic review and meta-Analysis. PLoS One. 2015;10(9):e0137444. doi:10.1371/journal.pone.0137444 26383245 PMC4575170

[20] Toyoda K, Yoshimura S, Nakai M, ; Japan Stroke Data Bank Investigators. Twenty-year change in severity and outcome of ischemic and hemorrhagic strokes. JAMA Neurol. 2022;79(1):61-69. doi:10.1001/jamaneurol.2021.4346 34870689 PMC8649912

[21] Weimer JM, Gordon E, Frontera JA. Predictors of functional outcome after subdural hematoma: a prospective study. Neurocrit Care. 2017;26(1):70-79. doi:10.1007/s12028-016-0279-1 27230968

[22] Poon MTC, Rea C, Kolias AG, Brennan PM; British Neurosurgical Trainee Research Collaborative (BNTRC). Influence of antiplatelet and anticoagulant drug use on outcomes after chronic subdural hematoma drainage. J Neurotrauma. 2021;38(8):1177-1184. doi:10.1089/neu.2018.6080 30526281 PMC8060161

[23] Gaist D, García Rodríguez LA, Hellfritzsch M, . Association of antithrombotic drug use with subdural hematoma risk. JAMA. 2017;317(8):836-846. doi:10.1001/jama.2017.0639 28245322

[24] Vora P, Pietila A, Peltonen M, Brobert G, Salomaa V. Thirty-year incidence and mortality trends in upper and lower gastrointestinal bleeding in Finland. JAMA Netw Open. 2020;3(10):e2020172. doi:10.1001/jamanetworkopen.2020.20172 33034641 PMC7547368

[25] Chornenki NLJ, Odabashiain R, Lenteejens J, Stucki F, Tritschler T, Siegal D. All-cause mortality after major gastrointestinal bleeding among patients receiving direct oral anticoagulants: a systematic review and meta-analysis. Blood. 2023;142(suppl 1). doi:10.1182/blood-2023-174340 PMC9749304 36514164

[26] Gómez-Outes A, Alcubilla P, Calvo-Rojas G, . Meta-analysis of reversal agents for severe bleeding associated with direct oral anticoagulants. J Am Coll Cardiol. 2021;77(24):2987-3001. doi:10.1016/j.jacc.2021.04.061 34140101

[27] Burström K, Johannesson M, Diderichsen F. A comparison of individual and social time trade-off values for health states in the general population. Health Policy. 2006;76(3):359-370. doi:10.1016/j.healthpol.2005.06.011 16214258

[28] Luengo-Fernandez R, Gray AM, Bull L, Welch S, Cuthbertson F, Rothwell PM; Oxford Vascular Study. Quality of life after TIA and stroke: ten-year results of the Oxford Vascular Study. Neurology. 2013;81(18):1588-1595. doi:10.1212/WNL.0b013e3182a9f45f 24107865 PMC3806919

[29] Teppo K, Airaksinen KEJ, Jaakkola J, . Trends in treatment and outcomes of atrial fibrillation during 2007–17 in Finland. Eur Heart J Qual Care Clin Outcomes. Published online December 20, 2022. doi:10.1093/ehjqcco/qcac086 PMC10627815 36542420

[30] Coyle M, Lynch A, Higgins M, . Risk of intracranial hemorrhage associated with direct oral anticoagulation vs antiplatelet therapy: a systematic review and meta-analysis. JAMA Netw Open. 2024;7(12):e2449017. doi:10.1001/jamanetworkopen.2024.49017 39630447 PMC11618459

[31] Zenodo. Accessed April 15, 2025. https://zenodo.org/records/13323365

[32] Toennis T, Bertaglia E, Brandes A, . The influence of atrial high-rate episodes on stroke and cardiovascular death: an update. Europace. 2023;25(7):euad166. doi:10.1093/europace/euad166 37345804 PMC10319778

[33] Ishiguchi H, Shimizu A, Ishikura M, . Association between atrial high-rate episodes and ischemic/major bleeding events in patients with a cardiac implantable electronic device—a 10-year, single-center historical cohort study. Circ J. 2021;85(8):1329-1337. doi:10.1253/circj.CJ-20-1269 33867407

[34] Joglar JA, Chung MK, Armbruster AL, 2023 ACC/AHA/ACCP/HRS guideline for the diagnosis and management of atrial fibrillation: a report of the American College of Cardiology/American Heart Association Joint Committee on Clinical Practice Guidelines. Circulation. 2023;149(1). doi:10.1161/CIR.0000000000001193 PMC11095842 38033089

[35] Van Gelder IC, Rienstra M, Bunting KV, . ESC Guidelines for the management of atrial fibrillation developed in collaboration with the European Association for Cardio-Thoracic Surgery (EACTS): developed by the task force for the management of atrial fibrillation of the European Society of Cardiology (ESC), with the special contribution of the European Heart Rhythm Association (EHRA) of the ESC. Endorsed by the European Stroke Organisation (ESO). Eur Heart J. 2024;45(36):3314-3414. doi:10.1093/eurheartj/ehae176 39210723

[36] Gaudino M, Braunwald E, Stone GW. Beyond the classic major cardiovascular event outcome for cardiovascular trials. Eur Heart J. 2024;45(44):4700-4703. doi:10.1093/eurheartj/ehae478 39082738

[37] MacLean S, Mulla S, Akl EA, . Patient values and preferences in decision making for antithrombotic therapy: a systematic review: Antithrombotic Therapy and Prevention of Thrombosis, 9th ed: American College of Chest Physicians Evidence-Based Clinical Practice Guidelines. Chest. 2012;141(2)(suppl):e1S-e23S. doi:10.1378/chest.11-2290 22315262 PMC3278050

[38] Alonso-Coello P, Montori VM, Díaz MG, . Values and preferences for oral antithrombotic therapy in patients with atrial fibrillation: physician and patient perspectives. Health Expect. 2015;18(6):2318-2327. doi:10.1111/hex.12201 24813058 PMC5810657

[39] Mahajan R, Perera T, Elliott AD, . Subclinical device-detected atrial fibrillation and stroke risk: a systematic review and meta-analysis. Eur Heart J. 2018;39(16):1407-1415. doi:10.1093/eurheartj/ehx731 29340587

[40] Langén V, Winstén AK, Airaksinen KEJ, Teppo K. Clinical outcomes of atrial fibrillation screening: a meta-analysis of randomized controlled trials. Ann Med. 2025;57(1):2457522. doi:10.1080/07853890.2025.2457522 39862317 PMC12161479

[41] Samsa GP, Matchar DB, Goldstein L, . Utilities for major stroke: Results from a survey of preferences among persons at increased risk for stroke. Am Heart J. 1998;136(4):703-713. doi:10.1016/S0002-8703(98)70019-5 9778075

