# Supplementary material for: Net Benefit of Anticoagulation in Subclinical Device-Detected Atrial Fibrillation
Source: JAMA Netw Open. 2025 May 2;8(5):e258461. doi: 10.1001/jamanetworkopen.2025.8461 (PMC12048845; doi:10.1001/jamanetworkopen.2025.8461)
Supplement: Supplement 1. — eTable. Model input parameters used in the sensitivity analyses for patients with CHA2DS2-VASc scores of less than 4, 4, and more than 4 eFigure 1. Structure in the Markov decision model cycle. eFigure 2. Results of the probabilistic sensitivity analysis considering the uncertainty in the treatment effects for stroke and bleeding [file jamanetwopen-e258461-s001.pdf]

Supplemental Online Content

Winstén AK, Langén V, Juhani KE, Teppo K. Net benefit of anticoagulation in subclinical device-detected atrial fibrillation. *JAMA Netw Open*. 2025;8(5):. doi:

eTable. Model input parameters used in the sensitivity analyses for patients with CHA2DS2-VASc scores of less than 4, 4, and more than 4
eFigure 1. Structure in the Markov decision model cycle.
eFigure 2. Results of the probabilistic sensitivity analysis considering the uncertainty in the treatment effects for stroke and bleeding

This supplemental material has been provided by the authors to give readers additional information about their work.

© 2025 Winstén AK et al. *JAMA Network Open*.

eTable. Model input parameters used in the sensitivity analyses for patients with CHA_2DS_2-VASc scores of less than 4, 4, and more than 4.

**CHA_2DS_2-VASc <4**	**Untreated rate per 100 patient-years**
Ischemic stroke	0.99 [a]
Major bleeding	1.11 [a]
Death	3.40 [b]
**Effect of anticoagulation**	**Relative risk**
Ischemic stroke	0.87 [c]
Major bleeding	1.27 [c]
**CHA_2DS_2-VASc =4**	**Untreated rate per 100 patient-years**
Ischemic stroke	0.93 [a]
Major bleeding	1.00 [a]
Death	3.40 [b]
**Effect of anticoagulation**	**Relative risk**
Ischemic stroke	0.63 [c]
Major bleeding	1.31 [c]
**CHA_2DS_2-VASc >4**	**Untreated rate per 100 patient-years**
Ischemic stroke	1.78 [a]
Major bleeding	1.18 [a]
Death	5.60 [b]
**Effect of anticoagulation**	**Relative risk**
Ischemic stroke	0.44 [c]
Major bleeding	1.48 [c]

All other parameters and the proportions of bleeding subtypes were consistent with those in the main analysis.

[a] Average nonanticoagulated rate of the NOAH and ARTESiA trials

[b] All-cause mortality in the NOAH trial (ARTESiA did not report mortality rates in different risk score categories)

[c] Derived from the ARTESiA trial data

© 2025 Winstén AK et al. *JAMA Network Open*.

eFigure 1. Structure in the Markov decision model cycle.

The model was run separately for patients with and without anticoagulation with different transition probabilities.

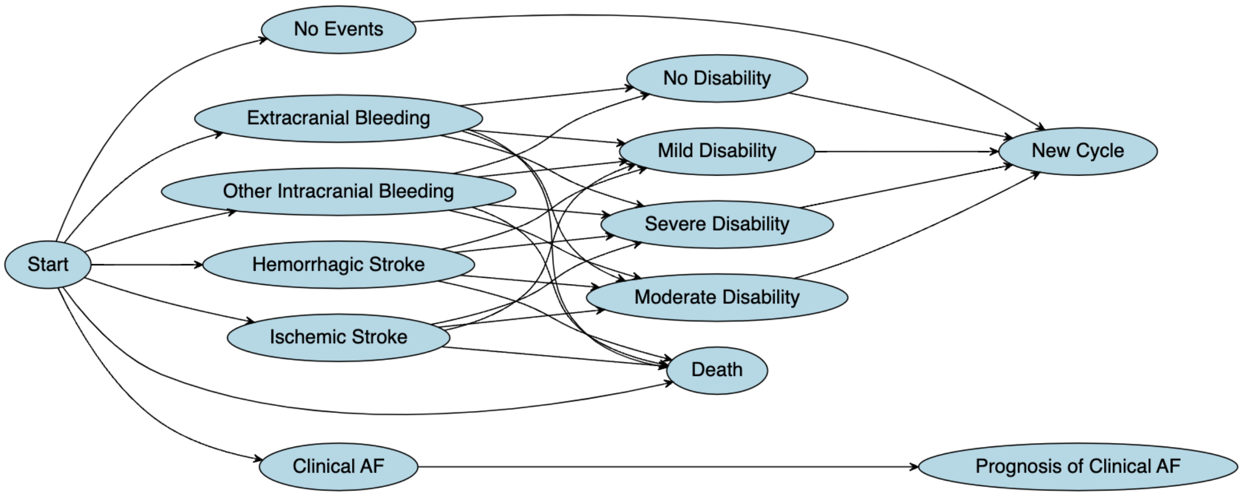

© 2025 Winstén AK et al. *JAMA Network Open*.

eFigure 2. Results of the probabilistic sensitivity analysis considering the uncertainty in the treatment effects for stroke and bleeding

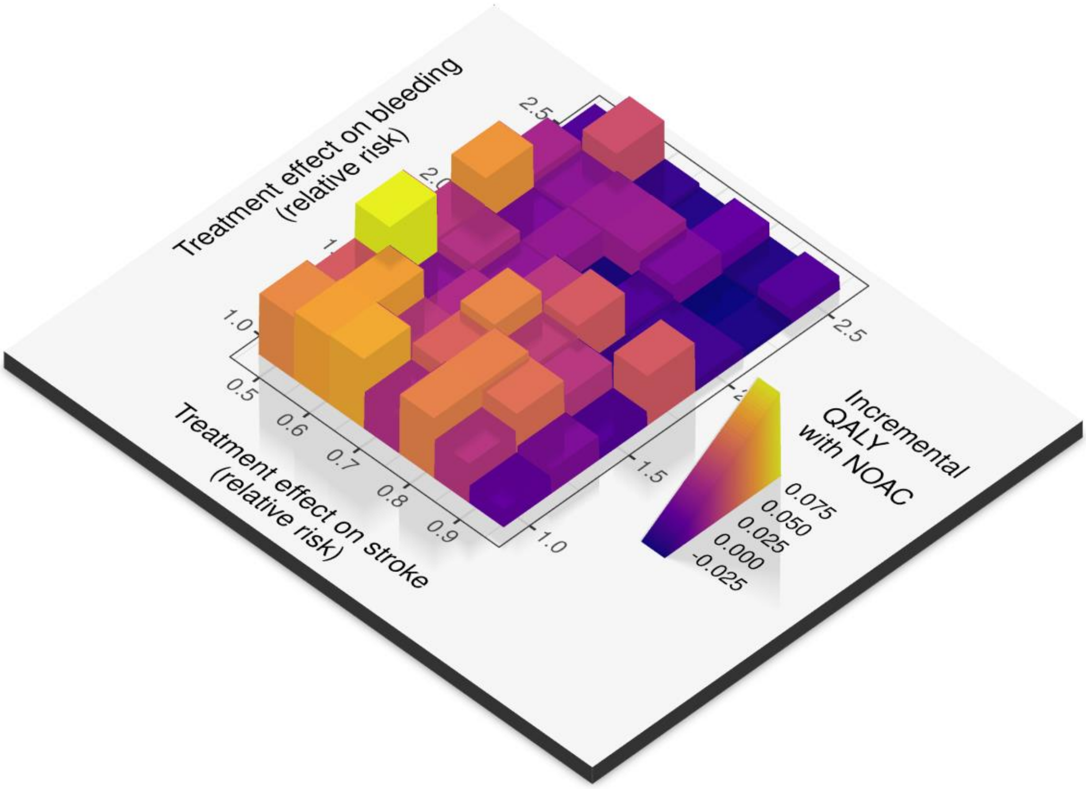

The figure displays incremental quality-adjusted life (QALYs) years per patient over a ten-year period with non-vitamin K antagonist oral anticoagulants (NOACs) within the range of the 95% confidence intervals of the relative risk estimates of treatment effects on stroke and bleeding. Brighter, higher areas and positive values indicate scenarios where starting anticoagulation led to an increase in QALYs, while darker, lower areas and negative values represent

scenarios with a reduction in QALYs with anticoagulation. The QALY difference corresponding to the heights and colors is illustrated in the legend.

© 2025 Winstén AK et al. *JAMA Network Open*.
