# Supplementary material for: Net Benefit of Anticoagulation in Subclinical Device-Detected Atrial Fibrillation
Source: JAMA Netw Open. 2025 May 2;8(5):e258461. doi: 10.1001/jamanetworkopen.2025.8461 (PMC12048845; doi:10.1001/jamanetworkopen.2025.8461)
Supplement: Supplement 2. — Data Sharing Statement [file jamanetwopen-e258461-s002.pdf]

Data Sharing Statement

Winstén. Net Benefit of Anticoagulation in Subclinical Device-Detected Atrial Fibrillation. *JAMA Netw Open*. Published May 02, 2025. doi:10.1001/jamanetworkopen.2025.8461

Data
Data available: Yes
Data types: Other (please specify)
Additional Information: In the interest of research reproducibility, we have deposited the codes of the Markov model in the Zenodo repository
How to access data: https://zenodo.org/records/13323365; DOI:10.5281/zenodo.13323365
When available: With publication

Supporting Documents
Document types: Statistical/analytic code
How to access documents: https://zenodo.org/records/13323365; DOI:10.5281/zenodo.13323365
When available: With publication

Additional Information
Who can access the data: For everyone
Types of analyses: All analyses
Mechanisms of data availability: With investigator support
